# Electrochemical Study of Semiconductor Properties for Bismuth Silicate-Based Photocatalysts Obtained via Hydro-/Solvothermal Approach

**DOI:** 10.3390/ma15124099

**Published:** 2022-06-09

**Authors:** Anastasiia V. Shabalina, Ekaterina Y. Gotovtseva, Yulia A. Belik, Sergey M. Kuzmin, Tamara S. Kharlamova, Sergei A. Kulinich, Valery A. Svetlichnyi, Olga V. Vodyankina

**Affiliations:** 1 Tomsk State University, Lenin Av. 36, Tomsk 634050, Russia; kara4578@mail.ru (E.Y.G.); belik99q@gmail.com (Y.A.B.); kharlamova83@gmail.com (T.S.K.); vodyankina_o@mail.ru (O.V.V.); 2 G.A. Krestov Institute of Solution Chemistry of the Russian Academy of Science, Akademicheskaja 1, Ivanovo 153045, Russia; smk@isc-ras.ru; 3 Research Institute of Science & Technology, Tokai University, Hiratsuka 259-1292, Kanagawa, Japan; skulinich@tokai-u.jp

**Keywords:** bismuth silicates, photocatalyst, semiconductor in liquid, interface, electrochemical study, electric double layer, zeta-potential

## Abstract

Three bismuth silicate-based photocatalysts (composites of Bi_2_SiO_5_ and Bi_12_SiO_20_) prepared via the hydro-/solvothermal approach were studied using electrochemical methods. The characteristic parameters of semiconductors, such as flat band potential, donor density, and mobility of their charge carriers, were obtained and compared with the materials’ photocatalytic activity. An attempt was made to study the effect of solution components on the semiconductor/liquid interface (SLI). In particular, the Mott–Schottky characterization was made in a common model electrolyte (Na_2_SO_4_) and with the addition of glycerol as a model organic compound for photocatalysis. Thus, a medium close to those in photocatalytic experiments was simulated, at least within the limits allowed by electrochemical measurements. Zeta-potential measurements and electrochemical impedance spectroscopy were used to reveal the processes taking place at the SLI. It was found that the medium in which measurements were carried out dramatically impacted the results. The flat band potential values (*E_fb_*) obtained via the Mott–Schottky technique were shown to differ significantly depending on the solution used in the experiment, which is explained by different processes taking place at the SLI. A strong influence of specific adsorption of commonly used sulfate ions and neutral molecules on the measured values of *E_fb_* was shown.

## 1. Introduction

Currently, catalysts based on nanostructured semiconductor materials are widely used for a variety of processes, ranging from organic chemical synthesis [1,2] to solar-induced hydrogen production [3]. So far, many efficient semiconductor photocatalytic systems have been developed or are in progress. Among them are bismuth-based nanomaterials that keep attracting more and more attention from researchers [4,5]. Bismuth silicates (Bi_2_SiO_5_, Bi_12_SiO_20_, Bi_4_Si_3_O_12_, etc.) and their composites seem to be promising photocatalysts [6,7,8,9], which is due to their activity in the visible range [10] and high polarization and internal electric field [11,12], as well as their ability to form effective junctions (S-scheme [13], type-II [14]), and so on. Previously, we obtained bismuth silicate (BSO)-based materials via mechanical activation [15] and laser fragmentation [16]. The latter material demonstrated catalytic activity in decomposing organic substances under visible light irradiation [16]. We also prepared photocatalytic BSO materials via a new hydro-/solvothermal approach and studied their activity for Rhodamine B (RhB) and phenol photo-decomposition [17].

The development of new photocatalysts would be unimaginable without studying carefully their morphology, chemical and phase composition, and drawing mechanisms of their interaction with target molecules under different conditions. The majority of photocatalytic reactions are carried out in liquids, e.g., degradation of water pollutants, oxygen or hydrogen evolution reactions, and so on. In this case, the catalytic process takes place at the semiconductor/liquid interface (SLI), which is formed when the semiconductor is immersed in a liquid. Several important processes are known to take place at the SLI, among which are: (1) charge transfer between semiconductor and components of the liquid; (2) formation of the electric double layer (EDL) consisting of a space charge (SC) layer in the semiconductor and the Helmholtz and Gouy layers located in the liquid; and (3) adsorption and formation of “surface states” [18]. Generally, when a semiconductor is placed into a liquid, the position of its conduction (CB) and the valence (VB) bands does not change, but its Fermi level (*E_F_*) moves towards the equilibrium state, resulting in the so-called “band bending” [19]. An equilibrium in the semiconductor/liquid system is achieved when the *E_F_* of the semiconductor and the *E_redox_* level of the solution are equalized. A flat band potential (*E_fb_*), at which the bands are flat, is a characteristic of such an equilibrium [20]. This is achieved using the SLI processes listed above. Therefore, investigating these processes is important for a better understanding of the effectiveness of the photocatalyst and the conversion mechanisms it is involved in.

Various electrochemical methods are commonly used to study SLI-related processes [21,22]. Voltammetry, photocurrent and/or open circuit potential measurements, electrochemical impedance spectroscopy (EIS), and other methods are applied to study the behavior of charge carriers and their participation in targeted photocatalytic processes. EIS occupies a special place in modern electrochemistry as one of its most informative and prospective analytic tools available. As a method, it reveals valuable information about electrochemical processes near the equilibrium state (and not only) of the studied system [23] (see Section S1, Supplementary Material).

Both EIS and calculations based on the Mott–Schottky (M–S) equation are actively used to reveal the semiconductor’s properties and mechanisms of processes occurring at its SLI. These approaches permit obtaining the *E_fb_* value and the concentration of charge carriers [19], both characteristics being necessary for further understanding of target photocatalytic processes, their effectiveness and mechanisms. Therefore, the above two techniques are widely applied to semiconductor-based systems, even though deviation from the M–S behavior is highly probable for a number of reasons, such as structural and compositional inhomogeneity of the material, appearance of surface states, and so on. [24,25]. The application of the EIS and M–S methods requires careful experimentation and extremely accurate handling of results. In this regard, clear and extensive recommendations can be found in the work of Hankin et al. [24].

Based on the above, this work aimed to obtain characteristics such as flat band potential, donor density, and mobility of charge carriers by applying electrochemical methods to three BSO-based photocatalysts prepared and described in our previous work [17]. The obtained results were then compared with the photocatalytic activity of the same samples, which allowed us to draw conclusions on the effect of solution components on the processes taking place at the SLI. Since the electrochemical parameters, such as *E_fb_*, are governed by components dissolved in the liquid, the mechanisms of photocatalytic decomposition of organic dyes are concluded to be studied electrochemically in the presence of the same organic molecules and in the same solvent that is used in photocatalytic experiments.

## 2. Materials and Methods

### 2.1. Reagents

A sodium silicate lump (Na_2_O × 3SiO_2_) was obtained from CheMondis GmbH (Köln, Germany), while the other reagents (TEOS, ethylene glycol, Bi(NO_3_)_3_, NaOH, Na_2_SO_4_, glycerol, polystyrene, 1,2-dichloroethane, HNO_3_, KCl, K_3_Fe(CN)_6_, and K_4_Fe(CN)_6_) were purchased from Merck (Germany) and used as received.

### 2.2. Synthesis of BSO Materials

All three BSO materials investigated in this work were obtained via the hydro-/solvothermal approach. Their preparation procedure, previously described elsewhere [17], is schematically presented in Figure S1. Below, the materials are denoted as BSO/TEOS, BSO/OH, and BSO/NaSi, since TEOS, NaOH and Na_2_Si_3_O_7_ were used as distinct reagents during their preparation, respectively (see Figure S1). In our previous work, these samples were marked as BSO_600, BSO_OH_600, and BSO_NaSi_OH_600, respectively [17].

### 2.3. Characterization of BSO Materials

Phase composition and size of coherent scattering regions (CSRs) were studied using an XRD 6000 diffractometer (Shimadzu, Japan) and the PDF-4 database. XPS analysis was performed with an ES-300 device (Kratos Analytical, Manchester, UK). The qualitative content of sample surfaces was studied by means of survey spectra, in accordance with the standard protocol, using the most intense spectral lines and taking into account the atomic sensitivity factors (ASF) for each element. The BET surface area was measured on the gas adsorption analyzer TriStar II 3020 (Micromeritics, Norcross, GA, USA). Before analysis, samples were degassed in vacuum (10^–2^ Torr) at 200 °C for 2 h. Scanning electron microscopy (SEM) images of electrode surfaces were obtained on a Vega 3H instrument (Tescan, Brno, Czech Republic) in back-scattered electron (BSE)–collecting mode.

### 2.4. Zeta-Potential Measurements

The zeta-potential of semiconductor particles was measured using an Omni S/N (Brookhaven, Holtsville, NY, USA) in ZetaPALS mode with a BI-ZTU autotitrator (Brookhaven, Holtsville, NY, USA). To measure zeta-potential values, samples were dispersed as powders (0.5 mg/mL) in distilled water or in a solution of interest: 1 mM aqueous Na_2_SO_4_, 1 mM aqueous glycerol, joint (1:1 molar ratio) aqueous solution of Na_2_SO_4_ (1 mM) and glycerol (1 mM). The pH values were adjusted by adding HNO_3_ solution.

### 2.5. Electrochemical Studies

#### 2.5.1. Electrochemical Measurements in Liquids

Electrochemical measurements in solutions were carried out using a CHI 660E electrochemical workstation (CH Instruments, Bee Cave, TX, USA). A three-electrode electrochemical cell was used, with a homemade graphite electrode (impregnated with polyethylene and paraffin) as a working electrode coated with semiconductor powder samples on its surface. A reference Ag/AgCl (1 M KCl) electrode and auxiliary Pt plate electrode were also applied. The electrodes were immersed in degassed (with Ar, 15 min) electrolyte solution that was placed in a sealed glass cell of 50 mL. Before proceeding to EIS and M–S measurements, linear sweep voltammograms in the region from −1 V to +1 V were recorded in each solution at a rate of 0.03 V/s.

The EIS measurements were carried out under a sinusoidal alternating voltage amplitude of 10 mV in the frequency range from 1 Hz to 100 kHz. The open circuit potential (OCP, *E_OC_*) was chosen as an initial *E* value for each measurement. The EIS data were analyzed and simulated using the ZView software (Scribner Associates Inc., Southern Pines, NC, USA).

M–S plots were registered using the impedance-potential mode of the CHI Electrochemical Station in the potential window chosen from voltammetry data at the frequency of 1 kHz. The capacity was calculated using *Z*” values [26] and was normalized to the geometric electrode area in accordance with standard protocols.

#### 2.5.2. Solutions for Electrochemical Measurements

All solutions used were prepared with distilled water (pH of 6.0, specific conductivity of 1.3 µS/cm, ion content below 3.2, 5.2, 0.6, and 20 µM for NO_3_^−^, SO_4_^2−^, Cl^−^ and Ca^2+^, respectively). To estimate the electroactive surface area of the working electrode (*S_EA_*), 0.025 M equimolar aqueous solution of K_3_Fe(CN)_6_ and K_4_Fe(CN)_6_ with 0.1 M KCl was used as a redox probe for cyclic voltammetry measurements. For the EIS measurements, two liquids were used: 0.5 M aqueous Na_2_SO_4_ (pH of 4.8) and a mixed aqueous solution (10:1 molar ratio) of 0.5 M Na_2_SO_4_ and 0.05 M glycerol (pH of 5.8).

#### 2.5.3. Preparation of Electrodes with BSO Material

The working graphite electrode was covered with a thick coating based on the studied material. For this, 18 mg of BSO powder was mixed with 2 mg of polystyrene and 100 μL of 1,2-dichloroethane. The obtained dispersion was sonicated for 3 min. Then, 5 μL of the obtained dispersion in polymer solution was applied to the graphite electrode’s surface and dried at room temperature for 12 h to get rid of the solvent. Thus, a relatively thick non-porous dielectric polymer coating covered the electrode surface, completely preventing any direct contact of the graphite electrode with the tested liquid. Hence, the entire electric contact was through the coating consisting of semiconductor particles dispersed in a polymer binder. For every measurement, at least three freshly prepared electrodes were used, after which the results were averaged, and accuracy was estimated.

#### 2.5.4. Electroactive Surface Area Measurements

To calculate the electroactive surface area (*S_EA_*), cyclic voltammetry measurements were carried out in the solution with a redox probe (K_3_/_4_Fe(CN)_6_), and voltammograms were recorded in the potential region from −1 V to +1 V at a scan rate of 0.03 V/s. The current of the anodic peak of the redox probe oxidation was measured, and *S_EA_* was calculated using the Rendles–Shevchik equation [27]:

*I* = 2.69 × 10^8^ *n*^3/2^ *S_EA_ D*^1/2^ *v*^1/2^ *C* (1)

where *I* is the peak current (in A); *D* is the diffusion coefficient (m^2^/s); *v* is the scan rate (V/s); *C* is the concentration of the redox probe (M); *n* is the number of the electrons involved in the oxidation process; and *S_EA_* expressed in m^2^.

#### 2.5.5. Measurements of Charge Carrier Mobility

A cell filled with a powder sample (so-called “solid electrochemical cell”) was used for the calculation of the mobility of charge carriers via the Mott–Gurney law [28,29]. It consisted of two flat steel electrodes (*d* = 5 mm). A powder sample was placed between the electrodes and fixed there with spring clamps with a constant force to a thickness of 1 mm (the mass of each material depended on its bulk density). The *I–V* curves (from 0 to +10 V) and PEIS data (eight potential steps at 0; 0.2; 1; 2; 3; 4; 6; and 8 V with 100 mV amplitude in the frequency range of 1–700 kHz) were registered using an SP150 potentiostat (BioLogi, Seyssinet-Pariset, France). The ZView (Scribner, New York, NY, USA) and EC-Lab (BioLogic, Seyssinet-Pariset, France) software were used for data simulation and calculations.

## 3. Results and Discussion

### 3.1. Characterization of BSO Samples

The phase composition and structural features (the size of coherent scattering regions, CSRs) obtained from XRD data are presented in Table 1. Two main phases of bismuth silicates, Bi_2_SiO_5_ and Bi_12_SiO_20_, are seen to be present in all the samples. Only sample BSO/TEOS was found to contain two additional oxide phases, α-Bi_2_O_3_ and β-Bi_2_O_3_. (The presence of a small amount of amorphous SiO_2_ at grain boundaries or between silicate layers was also possible in all the samples). Sample BSO/NaSi was found to have the highest content in the Bi_2_SiO_5_ phase. This sample also demonstrates the largest CSRs of 233 nm and, consequently, the lowest BET surface area among the three samples, i.e., 0.4 m^2^/g. The BET surface area of samples was found to decrease as follows: BSO/TEOS > BSO/OH > BSO/NaSi. However, the electroactive surface area values for both samples BSO/TEOS and BSO/OH were very similar, and the largest one was demonstrated by sample BSO/NaSi. In addition, Table 1 also presents the values of band gap energies (*E_g_*) obtained earlier for all three samples [17].

The XPS results on the relative content of Bi, Si, O, and Na found on the samples’ surfaces are presented in Table S1. The presence of trance amounts of Na found in two samples is explained by the use of NaOH during their preparation. Additionally, here, one should keep in mind that the surface may also contain a thin layer of SiO_2_, which could affect the results. Despite this, it is seen that the element ratio closest to that in Bi_2_SiO_5_ was observed for sample BSO/NaSi. The composition of its counterpart BSO/OH is seen to slightly deviate from the ratio of 2:1:5. Finally, sample BSO/TEOS demonstrated its surface composition to be the farthest from that of bismuth silicates. So, the XPS results are well consistent with those obtained by XRD.

The observed values of *S_EA_* are much lower than the geometric area of corresponding powders because of the working electrode’s design. The polystyrene layer covering the graphite surface only allowed for electric contact through the powder dispersed in it. Therefore, it was only the particles contacting the liquid that determined the *S_EA_*. This also explains the discrepancy between the BET and electroactive surface areas. The powder only partly contacted the solution being imbedded into the polystyrene binder, and the reduced contact area was a result of power pores filled with polymer.

**Figure 1:**
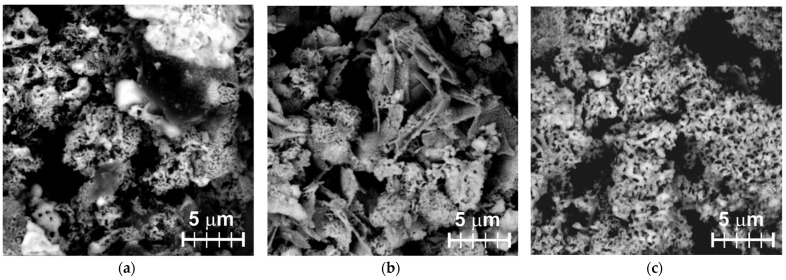
SEM images of samples recorded in BSE mode (Z-contrast): (**a**) BSO/TEOS, (**b**) BSO/OH, and (**c**) BSO/NaSi.

The surfaces of the prepared electrodes were characterized by SEM. Figure 1 presents SEM images obtained by collecting back-scattered electrons (in the so-called “*Z*-contrast” mode). In this case, larger atoms (i.e., with higher *Z*) are displayed as brighter spots in the image. Because of the polymer binder used, the electrode surfaces were smooth and pore-free (the BSO powders used were not porous, based on their low *S_BET_*). Thus, the bright spots seen in Figure 1 present BSO particles and their agglomerates, while the dark locations stand for polystyrene. Because of a relatively low binder fraction used, the dispersed BSO particles were in close contact with each other, providing good electric contact between the graphite electrode and tested liquid. It is seen in Figure 1 that all the working electrodes are characterized by a coral-like structure of their BSO material buried in polystyrene. The BSO/TEOS sample shows the most inhomogeneous structure, with denser and sparser areas (Figure 1a). Its BSO/OH counterpart in Figure 1b is seen to contain lamellar structures mixed with corals. The presence of denser and lamellar structures led to lower *S_EA_* values in these two samples. Finally, sample BSO/NaSi exhibits the most homogeneous distribution of small coral-like particles dispersed in the polymer (Figure 1c), which is why its *S_EA_* value was the largest.

### 3.2. Correlation between Electrochemically Determined Semiconducting Properties and Photocatalytic Activity of BSO Materials

**Figure 2:**
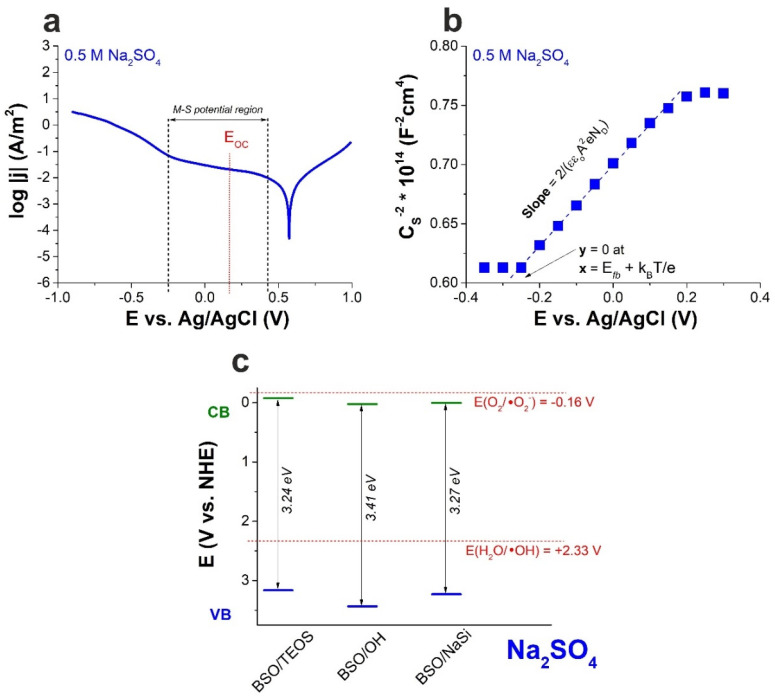
Results obtained by electrochemical methods for sample BSO/TEOS in 0.5 M aqueous Na_2_SO_4_: (**a**) Tafel representation of *I–V* curve; (**b**) Mott–Schottky plot; (**c**) Energy band diagram based on *E_fb_* values, with potentials of active radical formation marked with red (for ·OH and ·O_2_^−^ species).

To choose the region of potentials for the M–S measurements, corresponding *I–V* curves were obtained for the samples (Figure S2). The chosen regions were linear and located to the left of the turning point, as seen from the Tafel representation (as an example, see the case of sample BSO/TEOS in Figure 2a). This implies that the same mechanisms governed the processes at the SLI during M–S data collection. Additionally, the region without redox peaks was chosen for each sample in order to make sure that all the signals detected during measurements belonged to EDL and SC processes.

As mentioned above, the M–S technique is one of the most widely used methods for obtaining the *E_fb_* value. For all the samples, impedance was measured at different potentials in the chosen region at a fixed value of frequency (1 kHz), and *C_sc_* was determined for each potential. As a result, *C_sc_^−2^–E* curves were obtained (see an example in Figure 2b) with the slope direction characteristic of *n*-type semiconductors. The resulting *E_fb_* values calculated from the obtained plots for the three BSO samples are presented in Table 2. Measurements conducted in widely used Na_2_SO_4_ solution resulted in either positive (for samples BSO/OH and BSO/NaSi) or slightly negative (sample BSO/TEOS) *E_fb_* values.

Using the *E_fb_* values obtained in this work and the *E_g_* values provided in our previous work (Table 1), then taking that for *n*-type semiconductors *E_CB_* ≈ *E_fb_* [30] and assuming that the *E_fb_* for the materials was determined by a dominant phase (Bi_2_SiO_5_ for all the samples, see Table 1), we constructed energy band diagrams for the materials. The diagrams are shown in Figure 2c, clearly indicating that generating ·OH radicals is possible under photo-excitation of all the three materials, while their CB bottom is located too low for active ·O_2_^−^ radical formation.

Using the slope of the linear part of the M–S curve, donor density values were calculated following standard approach (Table 2). The mobility of charge carriers (*μ*) was also calculated, being within the range 30–45 cm^2^/V·s for all the samples (Table 2). Of all the samples, the BSO/TEOS one is seen to exhibit the highest mobility of its charge carriers even though its donor density was the lowest and its CB bottom is seen in Figure 2c to be the closest to the potential of active ·O_2_^−^ radical formation. Thus, sample BSO/TEOS appears to be the most promising material for photocatalytic applications.

The results of photocatalytic tests previously reported for the three samples are briefly given in Table 3. It should be mentioned that the photocatalytic activity of the materials was tested for 5 cycles, during which no significant decrease in their performance was observed. Table 3 shows that indeed sample BSO/TEOS exhibited the highest activity for both RhB and phenol degradation. Sample BSO/OH demonstrated quite close results, while sample BSO/NaSi was relatively inactive. This is well consistent with the values of charge carrier mobility obtained for the same samples tested in the solid electrochemical cell. However, according to the M–S measurements, the activity of all the materials (at least that of samples BSO/TEOS and BSO/OH) should have been much lower as they should not generate ·O_2_^−^ species. Hence, the question arises: why was their observed catalytic activity better than expected?

To answer this question, we considered all possible factors that could influence the results observed during either the electrochemical measurements or the photocatalytic process itself. One of the most obvious conditions that differed in the two above processes was the medium. More specifically, while the electrochemical measurements were carried out in quite a concentrated (0.5 M) solution of a strong electrolyte, the photocatalytic experiments were run in diluted solutions of organics (10^−5^ M). Therefore, it was decided to repeat similar electrochemical measurements in presence of organic molecules to see how they may affect the results.

In principle, both RhB and phenol can exhibit electrochemical activity. Rh B is known to undergo electrochemical oxidation, in particular in Na_2_SO_4_ solutions [31,32]. In turn, phenol exhibits electro-transformations and can form different products starting from 0.08 V [33,34]. That is why, to avoid additional electrochemical signals, we chose glycerol as another model organic substance that can only be electro-oxidized either at higher potential or in presence of complex electro-catalysts [35]. Being a polyol, aqueous glycerol is also used as a model sacrificial agent and a hole scavenger for photocatalytic hydrogen evolution processes [36,37]. Additionally, it is a non-electrolyte, thus exhibiting different nature and behavior than aqueous Na_2_SO_4_. Hence, we duplicated all electrochemical measurements in a joint Na_2_SO_4_-glycerol solution.

**Figure 3:**
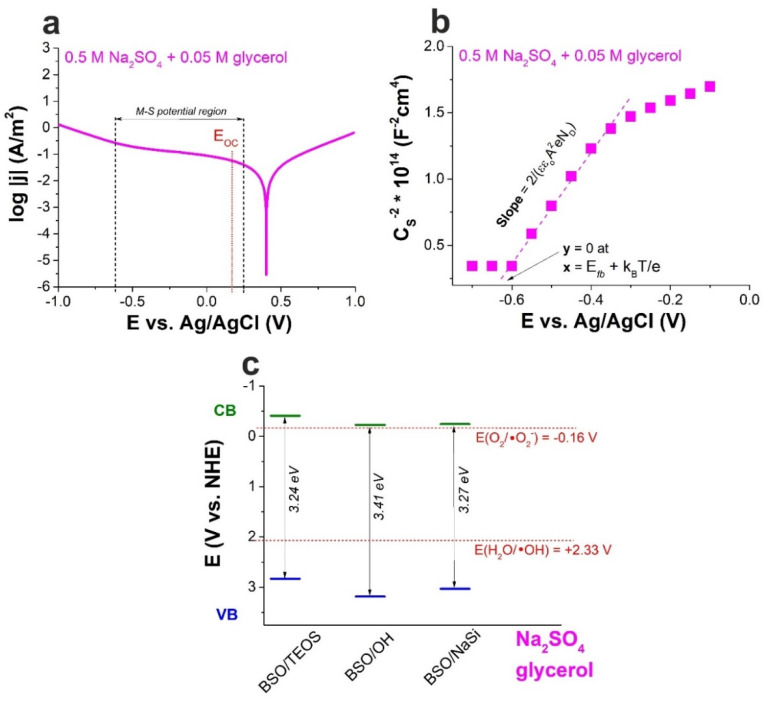
Results obtained by electrochemical methods for sample BSO/TEOS in 0.5 M aqueous Na_2_SO_4_ in presence of 0.05 M glycerol: (**a**) Tafel representation of *I–V* curve; (**b**) Mott–Schottky plot; (**c**) Energy band diagram based on *E_fb_* values (the potentials of active radical formation are marked with red).

The Tafel presentation of *I–V* curve and M–S plot obtained for sample BSO/TEOS in presence of glycerol are provided in Figure 3, while *E_fb_* values obtained for all the samples in presence of glycerol are listed in Table 4. It is seen that adding 0.05 M glycerol to the electrolyte lowered the *E_fb_* at least by 200 mV. Correcting the CB bottom location in Figure 2c using these values, one can find out that the formation of active oxygen radicals is possible for all the samples photoexcited in presence of glycerol (Figure 3c). Thus, *E_fb_* values obtained during electrochemical measurements are seen to be very sensitive to the medium used. However, is it the effect of presence of organic molecules/non-electrolyte, or just an experimental inaccuracy? Do the values obtained in joint solution describe the SLI state during photocatalytic experiments better? These are the questions to be answered in the following sections.

### 3.3. SLI State and Processes at SLI for BSO Materials in Two Media

The flat-band potential is one of the fundamental properties of any semiconductor–electrolyte system [38], and the Mott–Schottky technique is a common method for its determination. In fact, a lot of factors lead to deviations from ideal Mott–Schottky behavior, some of which were described, for example, by Cardon and Gomes [39]. Fynlason et al. measured flat-band potential values for three different forms of CdS (single crystal, thin film and powder) by three different methods, demonstrating some difference in obtained results [40]. Ge et al. also pointed out the high importance of the effect of surface states during *E_f_*_b_ studies [41].

Sharon and Sinha studied the effect of electrolytes on the flat-band potential of the n-BaTiO_3_ semiconductor [42]. They focused on the influence of pH and redox potential of electrolyte on the measured results and revealed that the negative flat-band potential increases with the increase in the redox potential of the electrolyte used. Additionally, for electrolytes containing Fe(CN)_6_^4−^ and I^−^ species, the specific adsorption of the anions was found to cause significant changes in Helmholtz potential, thus affecting flat-band potential values [42].

Typically, the majority of electrochemical measurements (including EIS and M–S measurements) are carried out in model electrolyte solutions. Sodium sulfate is one of such solutions [43,44,45], while KOH, H_2_SO_4,_ and phosphate-buffered solutions are also used (see, for example, [9,46,47]). However, when a semiconductor, which was characterized electrochemically in a Na_2_SO_4_ solution, is immersed in another liquid, the processes on its surface might change dramatically. Consequently, in another liquid medium, the resulting target process (e.g., photocatalytic hydrogen production or dye decomposition) might change its mechanism, slow down, or even completely stop. This implies that it is probably incorrect to describe and explain processes taking place in pure water or in the presence of sacrificial agent(s) via parameters obtained for the same semiconductor characterized in aqueous Na_2_SO_4_ (or another model electrolyte).

Sulfate anions are known to be prone to strong, specific, and spatially inhomogeneous adsorption [48] on semiconducting surfaces, even at large potential bias [49]. This leads to changes in EDL capacitance and surface charge and even affects the kinetics of electrochemical reactions at surfaces covered with SO_4_^2−^ [50]. Therefore, we suggest that the SLI of a semiconducting material should be electrochemically characterized in the same solution where it is used as a catalyst. So far, there were few works dealing with the liquids used in the present study, such as water [51], glycerol [37], and tris-HCl [52]. To the best of our knowledge, no systematic experimental work was carried out to compare EIS and M–S data of semiconductors characterized in liquids that contained components of photocatalytic media, and only a few relevant reports could be found. For example, a decreased accuracy of *E_fb_* determined in the presence of a hole scavenger (i.e., H_2_O_2_) was observed by Hankin and co-workers [24].

In the present work, we used the EIS method and zeta-potential measurements to study the difference in SLI state for the same BSO samples characterized in two solutions: model electrolyte (aqueous Na_2_SO_4_) and joint solution of Na_2_SO_4_ and model organic compound (glycerol) that is used in photocatalytic processes. For more information, the zeta-potential was also measured in water and glycerol solutions.

#### 3.3.1. Electrochemical Impedance Spectroscopy Studies

**Figure 4:**
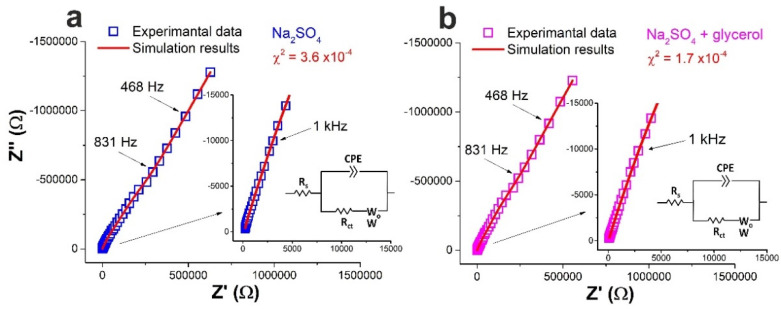
Typical Nyquist plots and simulated curves for sample BSO/TEOS in two liquids: (**a**) Na_2_SO_4_ solution; (**b**) joint Na_2_SO_4_ and glycerol solution. Corresponding equivalent circuits are given in insets.

A brief overview of some EIS studies on semiconductors characterized in different liquid media is given in Section S2 (Supplementary Material). Our results obtained for all samples were apparently quite similar. Before EIS measurements, LSV data were analyzed. Figure S2 demonstrates rectifying behavior of all the materials with classic current-potential curves characteristic of *n*-type semiconductor in the dark [53]. Then, EIS measurements at open circuit potentials listed in Table 5 were carried out. Typical EIS data, along with simulation results, obtained for sample BSO/TEOS in two liquids are presented in Figure 4.

As a next step, processes occurring at the SLI in two media were simulated for all the three samples, for which modified Randles equivalent circuits were constructed to fit the data observed both in aqueous Na_2_SO_4_ and Na_2_SO_4_ with glycerol [54,55]. The curves simulated using the ZView software showed good agreement with the experimental data, with *χ*^2^ factors being on the order of 10^−4^. Detailed parameters of all circuit elements are summarized in Table 6.

When the *CPE-P* value is between 0.8 and 1, the *CPE-T* characterizes a capacitance of EDL (*C_dl_*) [56]. At the series connection of *CPE* and *R_s_* elements, the value of *C_dl_* can be defined by the following equation [57,58]:

*C_dl_* = [*A_dl_* × *R_s_*^−(*α*−1)^]^1/*α*^ (2)

where *C_dl_* is the double-layer capacitance of the electrode/electrolyte surface; *A_dl_* is a parameter of the constant phase element (*CPE-T*); *R_s_* is the electrolyte resistance; and *α* is the *CPE-P* parameter [59]. Thus, the *C_dl_* values are also provided in Table 6.

The electrical double layer (EDL) is an important part of SLI. This section will discuss the EDL capacitance and composition, while results on two other components of the EIS circuit (solution resistance and diffusion) are discussed in Supplementary Material (Sections S5 and S6).

The EDL is known to be characterized by the thickness of its dense and diffuse layers. The diffuse part is solely determined by the electrolyte itself, while the dense part is influenced by adsorption phenomena. Shimizu and co-authors studied ion adsorption on the hematite surface using EIS [60], revealing that the specific adsorption of ions modified the surface’s structure and affected the EDL’s thickness. It is known that SO_4_^2−^ ions tend to specifically adsorb on semiconducting oxides, including bismuth oxide [61]. Some other ions possibly present in the water used in our experiments (NO_3_^–^, SO_4_^2−^, Cl^–^ and Ca^2+^) might also be prone to adsorption. Commonly, specific adsorption decreases the EDL capacitance.

As Table 6 shows, the double-layer capacitance of BSO-covered electrodes was in the range of 10^−5^ ÷ 10^−6^ F and generally differed for aqueous Na_2_SO_4_ medium with and without glycerol added. Only sample BSO/OH showed similar values of *C_dl_* for the two liquids tested. For sample BSO/TEOS, the EDL capacitance decreased about one order of magnitude in the presence of glycerol. On the contrary, for sample BSO/NaSi, its *C_dl_* increased one order of magnitude when glycerol was added. To explain these diverse data obtained for different samples, we addressed the electro-kinetic properties obtained from zeta-potential measurements and analysis.

#### 3.3.2. Results of Zeta-Potential Measurement

To reveal the specificity of the surface state of studied materials in the presence of solution components, we studied the zeta-potential and its change for all the samples in four different liquids. First of all, we used pure water to find out the surface state of the materials in the absence of other components. Then, we tested the most common electrolyte (Na_2_SO_4_), which is normally used for electrochemical studies, including those of *E_fb_*. Then, tests were run in a joint solution of Na_2_SO_4_ and glycerol to reveal the difference. Finally, we also tested how glycerol alone influences the surface state of the materials.

**Figure 5:**
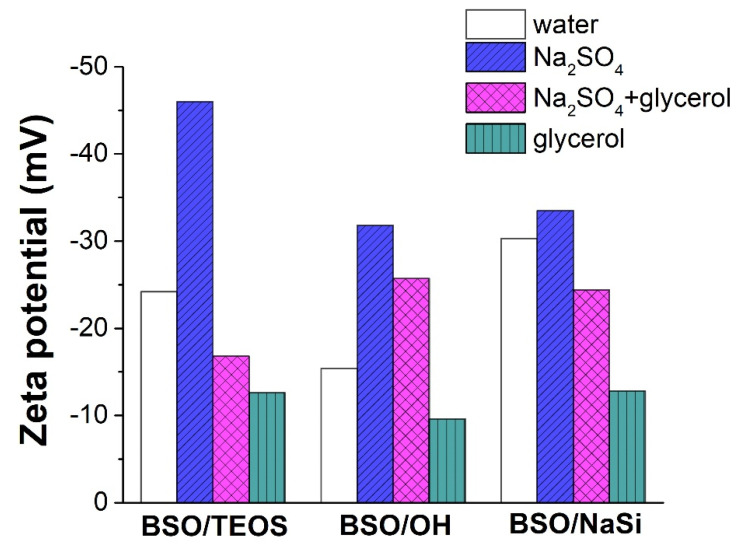
Results on zeta-potential measurement in four different liquids.

The obtained zeta-potential values measured in the above-mentioned media are exhibited in Figure 5 and Table S2, and those obtained at different pH are presented in Figure S3. All the materials demonstrated quite similar results, which indicates their similar nature.

As clearly seen in Figure 5 and Table S2, the most negative and the largest by their absolute value zeta-potentials were observed in the aqueous Na_2_SO_4_ medium. For samples BSO/TEOS and BSO/OH, their values were found to be almost twice larger than in water (compare blue and white bars in Figure 5). At the same time, the least negative and the lowest by absolute value zeta-potentials were detected in glycerol solution (see green bars in Figure 5). More details can be found in Section S3 (Supplementary Material).

Thus, it is obvious that the electro-kinetic properties of the studied BSO materials are affected by the surrounding liquid. Hence, depending on the medium chosen for the photocatalytic process, the catalyst may exhibit quite a different surface state. It is also possible that some components of the liquid medium can either increase or decrease material’s activity, even though they do not take part in the process themselves (unlike, for example, hole scavengers which do participate in the photocatalytic process).

When the studied BSO materials are moved from one solution to another, obviously, the composition of the adsorbed surface layer changes. At different pH values, the ratio of species absorbed on the surface varies as a result of their electric and other properties. Therefore, changes in pH lead to a gradual replacement of one type of surface molecule with others. The extrema in the zeta-potential–pH curves seen in Figure S3 are believed to indicate structural rearrangements of the adsorption layer. For convenience, Figure S4 schematically presents the surface composition of different liquids.

The BSO surface is positively charged (*n*-type semiconductor) when in contact with the aqueous solution. This positive charge is known to be located at the Bi^3+^, [Bi_2_O_2_]^2+^ or other surface centers and interacts with ions from the solution. While more details and reactions expected on the surface are provided in Section S4 (Supplementary Materials), below, we give a short account of our findings.

The EDL formed in water should consist of relatively small single-charged ions (see Figure S4). In the glycerol environment, the zeta-potential is expected to be more positive. In this case, the polyol can be adsorbed through the interaction with positively charged surface centers [62,63]. As a result, polyol functional groups become potential-determining species. Then, because glycerol molecules do not dissociate (as a non-electrolyte) and are quite large, the negative surface charge of the BSO particles in glycerol solution decreases in comparison with the same BSO particles immersed in pure water (Figure 5). The zeta-potential decreased when the samples were immersed in Na_2_SO_4_ solution, and the low IEP values might be connected with the surface’s recharging, which must be caused by SO_4_^2−^ anions adsorbed on positively charged surface sites (Figure S4).

It is thus seen that the presence of large double-charged ions on the electrode’s surface increases the *C_dl_* values for both solutions of Na_2_SO_4_ and Na_2_SO_4_ with glycerol. Hence, the results obtained for two-component solutions point to competitive adsorption of Na_2_SO_4_ and glycerol with sulfate anions in a leading position (Figure S4).

Thus, in the case of water-glycerol dispersions, zeta-potential could probably change due to the adsorption of polyol molecules and ions from water. In contrast, the results obtained in aqueous Na_2_SO_4_ and in two-component solutions can be explained by the semiconductor surface that only interacts with electrolytes, which normally increases *C_dl_* values.

So, the electrochemical measurements in solutions containing Na_2_SO_4_ characterize not the semiconductor’s surface itself but the SLI containing specifically adsorbed sulfate anions that affect EDL’s composition and capacity. That is why, when photocatalytic experiments are carried out, the SLI is different.

#### 3.3.3. Discussion and Findings

The SLI can be presented by two capacitors—*C_H_* and *C_sc_*—connected in a series [64]. The *C_H_* component stands for the capacitance of the Helmholtz layer in the electrolyte, and the *C_SC_* is the semiconductor’s space charge capacitance. In the case we investigated, *C_H_* >> *C_SC_* was ensured by the adsorption of anions/negatively charged fragments of molecules in the solution and on the surface of the studied sample (see Section S4). Thus, it is assumed that *C_dl_* ≈ *C_SC_*, and generally, the M–S approach can be used to study the semiconductor’s properties in the systems under investigation.

Specific adsorption plays an important role in processes occurring at the SLI since it affects the thickness of the dense EDL part. Large neutral molecules of glycerol provide a thicker layer than smaller SO_4_^2−^ ions. So, we suggest that the difference in the *E_fb_* and *N_D_* values measured in different liquids could be caused by the specific adsorption of glycerol and/or sulfate anions, which immediately leads to changes in the SLI.

Getting back to the zeta-potential values (see Figure 5 and Table S2), it is seen that the lower the surface charging (i.e., the closer the surface to its neutral state), the larger potential should be applied to achieve flat-band conditions (the case of two-component Na_2_SO_4_-glycerol solution). Additionally, vice versa, when the surface charge is maximal (due to SO_4_^2−^ anion adsorption), a smaller negative potential shift is required to stop net charge transfer. Thus, specific adsorption of ionic or neutral species is seen to dramatically affect the measured *E_fb_* values.

So, obviously, the presence in the solution of a strong electrolyte, non-electrolyte, or both affected the *E_fb_* values obtained during electrochemical measurements. All of the processes taking place at the SLI contributed to the semiconductor’s surface state, also influencing the characteristics of the material during its analysis. Consequently, it is believed that during photocatalytic tests, the results were affected not only by the absence of strong electrolyte but also by changes in the semiconductor’s surface charge (in the presence or absence of ions or large molecules of organics, dyes, etc.) and adsorption processes (that influence the SLI composition). Thus, to better understand the mechanism of any photocatalytic process, the semiconductor–liquid characteristics should be studied not only in a model solution but also in the presence of other components involved (such as target contaminants, sacrificial agents, etc.).

### 3.4. Difference in EDL for Three BSO Samples

According to Figure 5 and Table S2, the BSO/NaSi sample exhibited the highest zeta-potential in water and the smallest increase in its negative potential when Na_2_SO_4_ was added (an increment of 10% vs. 90–100% for the other samples). In addition, it showed the highest potential increment when glycerol was added to water, with about a 60% decrease in its negative potential. Therefore, sample BSO/NaSi was more strongly affected by glycerol’s presence than by Na_2_SO_4_’s presence. This could be a reason why the *E_fb_* value obtained in aqueous Na_2_SO_4_ described its photocatalytic activity quite well. For the same reason, sample BSO/NaSi exhibited the lowest photocatalytic activity: together with low charge carriers’ mobility, its SLI state is much affected by the presence of organic molecules.

To understand the reasons for unexpected behavior observed for sample BSO/NaSi, one should investigate better the nature of this sample. Its phase composition is quite close to that of sample BSO/OH, but the content of phase Bi_2_SiO_5_ in sample BSO/NaSi is the highest among the three materials, while the content of phase Bi_12_SiO_20_ is minimal. This combination could explain the different behavior demonstrated by the electrode coated with sample BSO/NaSi in glycerol-containing media.

Moreover, according to the SEM images, the BSO/NaSi powder was homogeneously distributed in polystyrene and, consequently, on the electrode’s surface. Even though it demonstrated the lowest BET surface area (measured for its powder), it exhibited the highest electroactive surface area (measured for its electrode). This could explain why it adsorbed glycerol quantitatively better, which affected its zeta-potential, EDL, as well as processes occurring on its electrode surface.

## 4. Conclusions

Three nanomaterials based on bismuth silicates obtained via a hydro-/solvothermal approach were studied using electrochemical methods (voltammetry and electrochemical impedance spectroscopy) both in liquid-containing and solid electrochemical cells. Such characteristic parameters as flat-band potential, donor density and mobility of charge carriers were obtained for all the materials. The obtained results were compared with materials’ photocatalytic activity, revealing certain inconsistencies. In particular, according to the *E_fb_* values obtained in aqueous Na_2_SO_4_, the observed photoactivity of the materials was better than it was expected.

Thereafter, we attempted to simulate the medium used in photocatalytic experiments, at least within the limits that are acceptable for electrochemical measurements. For that, we added a model organic substance (glycerol) to the model electrolyte and obtained dramatically different values of *E_fb_*. Zeta-potential and EIS measurements were also carried out in the presence and in the absence of glycerol. Based on the obtained results, we suggest that two aspects should be taken into account when semiconductor photocatalysts are studied by electrochemical methods. On one hand, correct data can only be obtained in a liquid with a strong electrolyte from the experimental setup point of view since high ionic conductivity is required for high accuracy. On the other hand, however, the specific adsorption of ions (SO_4_^2−^ and/or others) and other species (organic molecules, surfactants, etc.) present in electrolytes may affect the processes occurring at the SLI. Consequently, conclusions on the semiconductor’s behavior drawn from systems’ “electrode-liquid” with model solutions will not necessarily be applicable to systems with “real” liquids used for photocatalytic processes. The presence of components of interest (the target contaminants, sacrificial agents, etc.) during the photocatalytic process and their influence on the SLI should be taken into account.

## Figures and Tables

**Table 1:** Composition and structural characteristics of BSO samples.

Sample	Phase Composition (Content, %) ^a^	Structural Features ^b^, nm	BET Surface Area ^a^, m^2^/g	Electroactive Surface Area ^c^, cm^2^	*E_g_*^a^, eV
BSO/TEOS	Bi_2_SiO_5_ (79) Bi_12_SiO_20_ (16) α-Bi_2_O_3_ (3) β-Bi_2_O_3_ (2)	150	12 ± 2	(3.2 ± 0.1) × 10^−4^	3.24 2.81 – 2.10
BSO/OH	Bi_2_SiO_5_ (85) Bi_12_SiO_20_ (15)	227	2.0 ± 0.4	(3.24 ± 0.08) × 10^−4^	3.41 3.02
BSO/NaSi	Bi_2_SiO_5_ (96) Bi_12_SiO_20_ (4)	233	0.40 ± 0.08	(13.0 ± 0.9) × 10^−4^	3.27 2.88

^a^ Data previously published elsewhere [17]. ^b^ CSR values obtained from XRD data were used to characterize the microstructure of the main phase in the materials (Bi_2_SiO_5_). ^c^ Geometric area of the working electrode was 0.28 cm^2^_._

**Table 2:** Data from electrochemical measurements in aqueous Na_2_SO_4_ (*E_fb_* and *N_D_*) and for powder samples (*µ*).

Sample	*E_fb_*^a^, V vs. NHE	*N_D_*^a^, m^−3^	*μ*, cm^2^/V·s
BSO/TEOS	–0.076 ± 0.008	10^22^	45
BSO/OH	+0.024 ± 0.009	10^23^	43.9
BSO/NaSi	+0.004 ± 0.001	10^23^	30.2

^a^ From M–S results obtained in 0.5 M Na_2_SO_4._

**Table 3:** Photocatalytic activity of the BSO samples from [17].

Sample	Photocatalytic Conversion ^a^, %		
	Rhodamine B		Phenol
	Xe ^b^	LEDs ^c^	LEDs ^c^
BSO/TEOS	88	100	28
BSO/OH	75	100	27
BSO/NaSi	52	66	7

^a^ For a period of 4 h. ^b^ Full spectrum. ^c^ Wavelength of 378 nm.

**Table 4:** Flat band potential values for BSO samples measured in presence of glycerol.

Liquid	*E_fb_* (Mott–Shottky), V, vs. NHE		
	BSO/TEOS	BSO/OH	BSO/NaSi
Glycerol and Na_2_SO_4_ in H_2_O	−0.409 ± 0.006	−0.225 ± 0.004	−0.242 ± 0.001

**Table 5:** OCP values for electrodes with BSO samples in two media.

Liquid	*E_OC_*, V, vs. Ag/AgCl		
	BSO/TEOS	BSO/OH	BSO/NaSi
Na_2_SO_4_ in H_2_O	+0.17 ± 0.02	+0.170 ± 0.005	+0.16 ± 0.02
Glycerol and Na_2_SO_4_ in H_2_O	+0.170 ± 0.005	+0.112 ± 0.002	+0.24 ± 0.03

**Table 6:** Fitting results for EIS experimental data of BSO samples tested in two liquids.

Sample		BSO/TEOS		BSO/OH		BSO/NaSi	
Liquid		Na_2_SO_4_	Na_2_SO_4_ Glycerol	Na_2_SO_4_	Na_2_SO_4_ Glycerol	Na_2_SO_4_	Na_2_SO_4_ Glycerol
Parameters	*R_s_*_,_ Ω	130	20	50	50	30	30
	*CPE-T*, Ω^−1^s*^α^*	4 × 10^−8^	5 × 10^−8^	9 × 10^−8^	4 × 10^−8^	2 × 10^−8^	2 × 10^−7^
	*CPE-P* (*α*)	0.85	0.84	0.86	0.85	0.90	0.81
	*R_ct_*_,_ Ω	40	30	20	30	40	20
	*C_dl_*, F	5 × 10^−5^	9 × 10^−6^	4 × 10^−5^	2 × 10^−5^	4 × 10^−6^	5 × 10^−5^
	*Wo-R*, Ω	8 × 10^5^	5 × 10^5^	2 × 10^6^	8 × 10^5^	1×10^6^	1 × 10^6^
	*Wo-T*, s	9 × 10^−3^	2 × 10^−3^	5 × 10^−2^	2 × 10^−2^	4×10^−3^	4 × 10^−3^
	*Wo-P*	0.29	0.28	0.18	0.35	0.28	0.69
	*χ* ^2^	3.6 × 10^−4^	1.7 × 10^−4^	2.3 × 10^−4^	3.0 × 10^−4^	4.5×10^−4^	9.6 × 10^−4^

## Supplementary Materials

The following supporting information can be downloaded at: https://www.mdpi.com/article/10.3390/ma15124099/s1, Section S1: EIS theory concise basics; Figure S1: Scheme of synthesis for the BSO nanomaterials used; Table S1: Relative content of elements on sample surfaces according to XPS analysis; Figure S2: LSVs for the electrodes with the BSO samples in two liquids; Section S2: An overview of some results of EIS study of semiconductors in different solutions; Section S3: More details on the results of electro-kinetic properties study; Figure S3: Change in zeta-potential values as the colloids’ pH increased; Table S2: Zeta-potential values of the particles of BSO samples in different media; Section S4: Surface composition discussion; Section S5: Solution resistance study; Section S6: Diffusion process at the SLI.

## Abbreviations and Designations

BSO	bithmuth silicon oxides (bismuth silicates)
RhB	Rhodamine B
SLI	semiconductor/liquid interface
EDL	electric double layer
SC	space charge layer
CB	conduction band
VB	valance band
*E_F_*	Fermi level
*E_redox_*	redox potential of the electrolyte
*E_fb_*	flat band potential
EIS	electrochemical impedance spectroscopy
M–S	Mott–Schottky
CSRs	coherent scattering regions
SEM	scanning electron microscopy
BSE	back-scattered electrons
OCP, *E_OC_*	open circuit potential
*S_EA_*	electroactive surface area
TEOS	tetraethoxysilane
XRD	X-ray diffraction
*E_g_*	band gap energy
*S_BET_*	specific surface area according to BET
NHE	Normal Hydrogen Electrode
*N_D_*	donor density
*μ*	mobility of charge carriers
LEDs	light-emitting diodes
*C_dl_*	EDL capacitance
*C_H_*	Helmholtz layer capacitance
*C_SC_*	space charge layer capacitance
CPE-T, Rs, and other EIS simulation parameters.	are described in Section S1 (Supplementary Material).
IEP	isoelectric point
