# Supplementary material for: Electrochemical Study of Semiconductor Properties for Bismuth Silicate-Based Photocatalysts Obtained via Hydro-/Solvothermal Approach
Source: Materials (Basel). 2022 Jun 9;15(12):4099. doi: 10.3390/ma15124099 (PMC9229303; doi:10.3390/ma15124099)
Supplement: Supplementary file 1 [file materials-15-04099-s001.zip › materials-1708074-supplementary.pdf]

Supplementary Material for

Electrochemical Study of Semiconductor Properties for Bismuth Silicate-Based Photocatalysts Obtained via Hydro-/Solvothermal Approach

Anastasiia V. Shabalina[1,*], Ekaterina Y. Gotovtseva[1], Yulia A. Belik[1], Sergey M. Kuzmin[2], Tamara S. Kharlamova[1], Sergei A. Kulinich[3], Valery A. Svetlichnyi[1,*], Olga V. Vodyankina[1]

[1] Tomsk State University, Lenin av. 36, Tomsk 634050, Russia. kara4578@mail.ru (E.Y.G.), belik99q@gmail.com (Y.A.B.), kharlamova83@gmail.com (T.S.K.), vodyankina_o@mail.ru (O.V.V.)

[2] G.A. Krestov Institute of Solution Chemistry of the Russian Academy of Science, Akademicheskaja 1, Ivanovo 153045, Russia. smk@isc-ras.ru (S.M.K.)

[3] Research Institute of Science & Technology, Tokai University, Hiratsuka, Kanagawa 259-1292, Japan; skulinich@tokai-u.jp (S.A.K.)

[*] Corresponding authors. E-mail: shabalinaav@gmail.com (A.V.S.), v_svetlichnyi@bk.ru (V.A.S.)

Section S1. EIS theory concise basics.

A concise theoretical description of EIS is provided in this section.

The EIS method is used to determine the transport functions of a system under its excitation with a low amplitude signal in the form of a sinusoidal wave. The low amplitude of the signal makes it possible to consider the response as linear, so that the signal passed through the system under study changes its amplitude and phase, but the signal does not change its shape. Such signal changes can be described using impedance (*Z*), which is defined as the total resistance and is represented in a form of a complex number having two components–real (Z', active resistance) and imaginary (Z", reactive resistance) parts:

$$Z = Z' - jZ'' \quad , \qquad (2)$$

where j is an imaginary unit.

The studied system usually consists of an electrochemical cell with two (working and counter) or three (working, counter, and reference) electrodes that (for the wet systems) are immersed in a liquid electrolyte and are connected to an electrochemical workstation. The system under investigation is disturbed by an input sinusoidal signal (potential or current), and an output response (current or potential) is measured. We will focus on the sinusoidal disturbance of the potential hereafter.

The EIS method consists of the following steps:

1. The potential (E) of the working electrode in the system under study is set at an initial value (E_{init}). Then, E is programmatically perturbed around its value in a sinusoidal manner with a certain amplitude (E_{amp}), with the frequency (ω) changing step-by-step in a selected region.

2. The resulting sinusoidal current (I) flows through the investigated system.

3. Two parameters are measured as an output signal: the phase angle (φ) between the input potential and output current and the system's total impedance (Z).

Thus, the measured Z values are divided into two components by software. Then, the collected EIS results are represented in either Nyquist (Z'–Z'') or Bode (ω–Z or ω–φ) coordinates [*Mark E. Orazem, B. Tribollet, Electrochemical impedance spectroscopy, John Wiley & Sons, Inc., Hoboken, New Jersey, 2008.*]. The obtained experimental data is analysed using software (ZView, EIS Spectrum Analyser, ZSimpWin, MEISP, and others). Subsequently, the equivalent electric circuit is constructed via simulation, fitting, and verification of a physical meaning. Simulation expresses the kinetic parameters of electrochemical reactions and physical processes on the electrode through the electric circuits. The experimental data were fitted with the simulation results to provide the values of the parameters corresponding to the circuit elements. These elements and their locations in the circuit must possess a physical meaning. Thus, the creation of the equivalent circuit provides a connection between the physical/chemical and electrical parameters. The obtained circuit can help to reveal the mechanisms of the processes in the studied system [*A. Lasia, Electrochemical impedance spectroscopy and its applications, Springer, New York, 2014.*].

The equivalent circuit comprises individual elements, usually including the resistance, capacitance, constant phase element, Warburg impedance, etc. [*J. Brock, Electrochemical impedance spectroscopy. Methods, analysis and research, Nova, New York, 2017.*]. The resistance in EIS-equivalent circuits are usually attributed to charge transfer phenomena. This element's electrochemical meaning is the electrolyte's resistance, the resistance of a charge transfer through the electric double layer (EDL), the effective rate of electrochemical reaction, etc. Capacitance (C) simulates the accumulation of charge carriers at electrochemical interfaces and the accumulation or depletion of charge carriers in the semiconductor's space charge (SC) layer. In addition, this element simulates a delay of a process in relation to another one.

A constant phase element (CPE) is an element for simulating the impedance of a wide class of electrochemical processes. This element combines simulations of the impedance behavior of the electrochemical reaction caused by both the overcoming energy barrier during charge/mass transfer and the structure's inhomogeneity of the electrode surface [*J.-B. Jorcin, M. E. Orazen, N. Pebere, B. Tribollet, CPE analysis by local electrochemical impedance spectroscopy, Electrochim. Acta. 51 (2006) 1473–1479. https://doi.org/10.1016/j.electacta.2005.02.128.*]. *CPE* is described by two parameters: *CPE-P* and *CPE-T*.

E. Warburg introduced the Warburg element (*W*) to describe the impedance of ideal linear semi-infinite diffusion [*E. Warburg, Ueber das Verhalten sogenannter unpolarisirbarer Elektroden gegen Wechselstrom, Annalen deb physik und chemie. 67 (1899), 493–499. https://doi.org/10.1002/andp.18993030302.*]. The *W* element consists of three parameters: the diffusion time (*W-T*), the diffusion resistance (*W-R*), and a characteristic of the diffusion ideality (*W-P*). For an ideal diffusion process, the W-P parameter is 0.5 [*A. Doi, Comment on Warburg impedance and related phenomena, Solid State Ion. 40 (1990) 262–265. https://doi.org/10.1016/0167-2738(90)90336-P.*]. Deviation from this value means a deviation from the ideal diffusion nature for the process. This is due to the inhomogeneity of the composition and/or the electrode surface's structure.

Thus, the EIS experimental results are commonly represented as equivalent circuits with the fitted parameters' values. Within such an approach, the corresponding elements of the circuit can qualitatively and quantitatively describe the physical processes in the investigated system.

The Nyquist plot demonstrates both the area of kinetic control of the process (semicircle part) and the diffusion-controlled area (straight line with a single slope). The equivalent circuit contains a double-layer capacitance C_{dl} and uncompensated solution resistance R_s. The reaction resistance is characterized by the sum of the charge transfer resistance R_{ct} and Warburg impedance *W*. Oxidation and reduction processes occurring on the electrode's surface are associated with the diffusion delivery/removal of the ions to or from the surface. In this case, the Warburg impedance implies a series connection of resistance and capacitance [*V. F. Lvovich, Impedance spectroscopy. Applications to electrochemical and dielectric phenomena, John Wiley & Sons, Inc., Hoboken, New Jersey, 2012.*].

Thus, the EIS method provides information about the mechanisms and kinetics of the processes occurring on the electrode's surface without disturbing the nature of these processes. On the basis of the equivalent circuits, the elements can be interpreted according to the physical-chemical nature of the processes occurring in the system. Despite some subjectivity and ambiguity in the interpretation of the EIS data, the results are informative and illustrative, making this method very effective.

Figure S1.

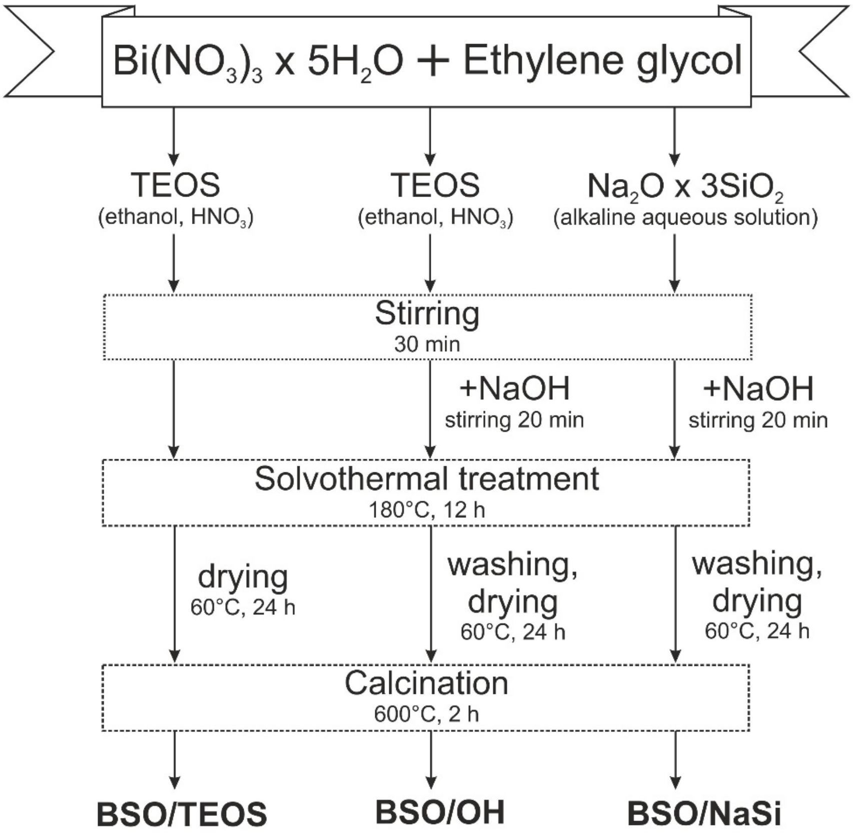

Figure S1 – Scheme of synthesis for the BSO nanomaterials used.

Table S1.

Table S1 – Relative content of elements on sample surfaces according to XPS analysis

Sample	Relative content of element				Approximate Bi:Si:O ratio
	Bi	Si	O	Na	
BSO/TEOS	1	3.6	7.9	0	2:8:16
BSO/OH	1	1.1	3.1	0.1	2:2:6
BSO/NaSi	1	0.6	2.6	0.2	2:1:5

Figure S2.

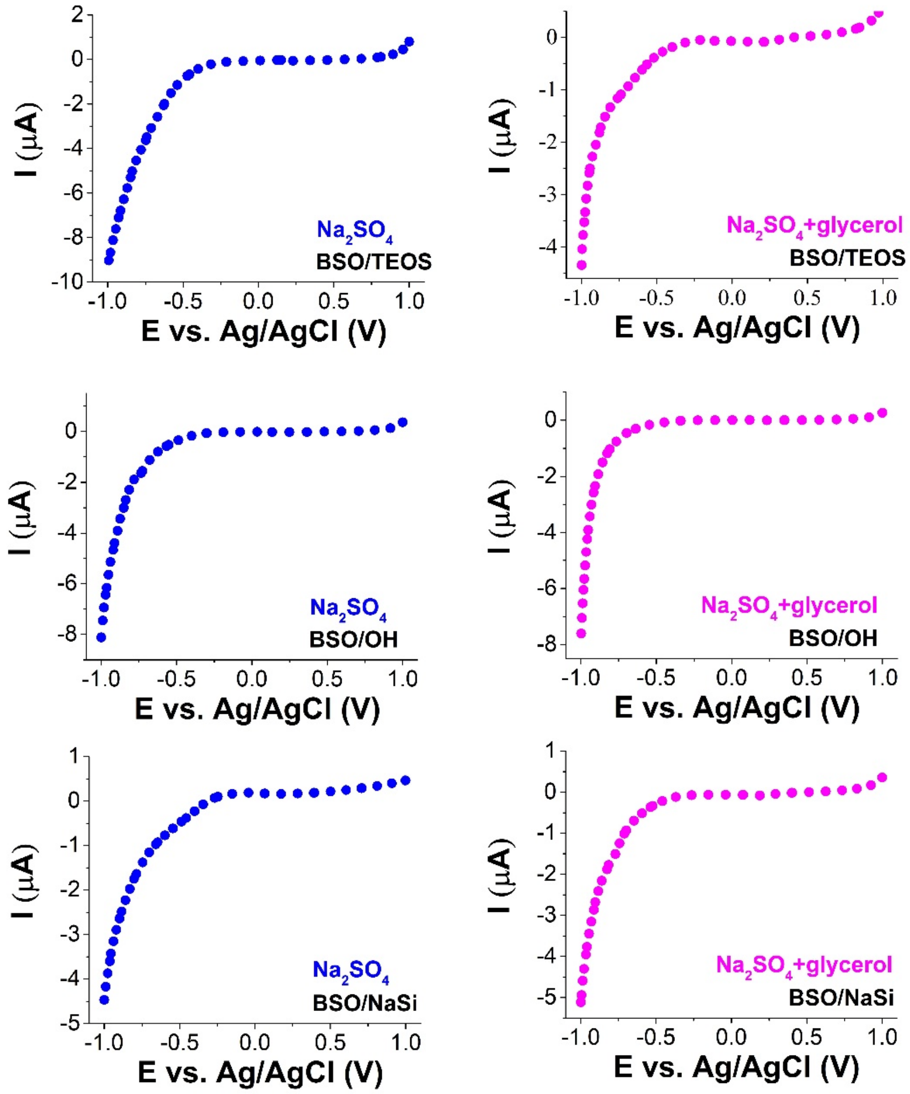

Figure S2 – LSVs for the electrodes with the BSO samples in two liquids: 0.5 M aqueous Na_2SO_4 (blue color) and mixed aqueous 0.5 M Na_2SO_4 and 0.05 M glycerol (pink color).

Section S2. An overview of some results of EIS study of semiconductors in different solutions.

The majority of EIS measurements for semiconductor electrodes are carried out in Na_2SO_4 solutions (see, for example [1-5]). Dai [6] used the classic Randles equivalent circuit R_s–(C||R_p) to simulate the processes on a passivating film of a FeCoCrNiMo alloy electrode. Si et al. [7] obtained a circuit containing both Randles and modified Randles circuits in a series for MoS_2/WSe_2 in sodium sulfate solution. Bashiri and co-authors [8] registered the impedance spectra for a TiO_2/Fe_2O_3 electrode in KOH solution in the presence of 5% glycerol. The circuit contained two parts, with one characterizing the charge transfer on the electrode/electrolyte interface and the diffusion process. Lopes et al. [9] obtained two-component circuit for Fe_2O_3 in NaOH solution. One component, consisting of the *CPE* and R_p, simulated the processes at the semiconductor's depletion layer, while the second part (*CPE*, *W*, and R_p) was attributed to the Helmholtz component of the electrode/electrolyte interface. Hwang [10] studied $BiVO_4$ films in a phosphate buffer solution in the presence of a hole scavenger (H_2O_2). The circuit they used to simulate the processes consisted of two Randles circuits in a series. Half of the second circuit was attributed to the solution component, while the rest of the circuit (excluding a part of R_s) simulated the processes on the photo-anode. Hence, analysis of the EIS data are interpreted differently, depending on the electrode system, material, solution, and the analyst's choice.

Section S3. More details on the results of electro-kinetic properties study

The initial colloids in distilled water were characterized by a pH between 5.7 and 5.9 and negative values for the particle's zeta-potential. A decrease in pH leads to an increase in the potential. The change of the zeta-potential sign is observed at pH values of 3–3.2.

The colloids in Na_2SO_4 demonstrated a similar nature of the potential's change with the pH (it increased as pH decreased). But the curves shifted towards lower pH values. Hence, the potential's sign changed at a pH of 1.7. The results obtained in the joint solution of sodium sulfate and glycerol are close to those registered in Na_2SO_4. The same shift towards lower pH was observed, and the surface charge changed at a pH of 1.7–2.3. The isoelectric point (IEP) values were close. However, when moving away from the IEP, the particles exhibited higher zeta-potentials at the same pH values in the presence of glycerol.

In glycerol solution, the zeta-potential increases as the pH decreases as well. However, the curves shifted towards higher pH values. Here, the surface charge changed to the opposite sign at a pH of 4.2–4.7.

Figure S3.

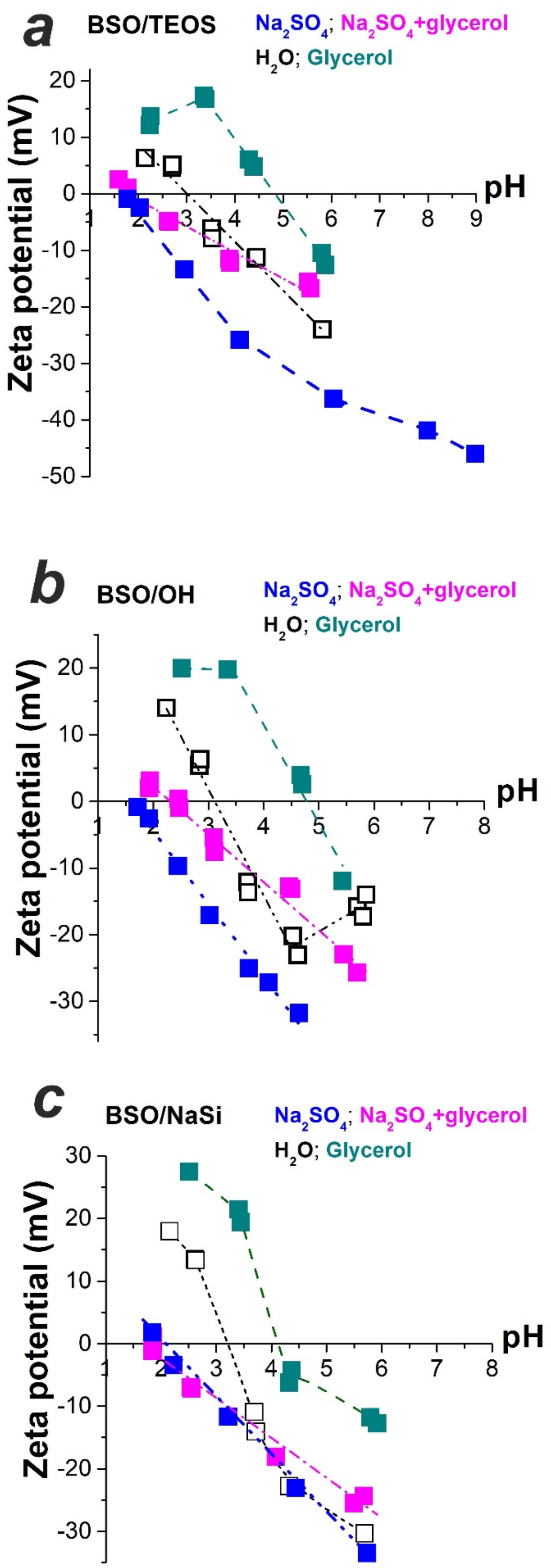

Figure S3 – Change in zeta-potential values as the colloids' pH increased: samples (a) BSO/TEOS, (b) BSO/OH, and (c) BSO/NaSi.

Table S2.

Table S2 – Zeta-potential values of the particles of BSO samples in different media.

Sample	Zeta-potential, mV			
	H_2O	Na_2SO_4	Na_2SO_4 glycerol	glycerol
**BSO/TEOS**	*–24.2*	*–46.0*	*–16.8*	*–12.6*
**BSO/OH**	*–15.4*	*–31.8*	*–25.7*	*–9.6*
**BSO/NaSi**	*–30.3*	*–33.5*	*–24.4*	*–12.8*

Section S4. Surface composition discussion.

The BSO surface is positively charged (*n*-type semiconductor) when in contact with the solution. This positive charge is located on the Bi^{3+}, $[Bi_2O_2]^{2+}$ sites or other surface centers and interacts with the ions from the solution.

One can suppose that in deionized water, the surface charge of BSO particles is determined by the functional groups formed from interactions with water molecules. At pH levels higher than the IEP, this process can be written as follows:

$$(=Bi\text{-}OH)_s + OH^- \leftrightarrow (=Bi\text{-}O^-)_s + H_2O$$

or

$$(=Si\text{-}OH)_s + OH^- \leftrightarrow (=Si\text{-}O^-)_s + H_2O$$

At a pH lower than the IEP, the interaction would change by:

$$(=Bi\text{-}OH)_s + H^+ \leftrightarrow (=Bi\text{-}OH_2^+)_s$$

or

$$(=Si\text{-}OH)_s + H^+ \leftrightarrow (=Si\text{-}OH_2^+)_s$$

Thus, the EDL formed in water should consist of relatively small single-charged ions. However, the water used in this work was not free from ions. As mentioned before, it contained NO_3^-, SO_4^{2-}, Cl^- and Ca^{2+}. They could also adsorb on the surface and contribute to its charge. Since their concentration was low (much lower than that of Na_2SO_4), their contribution was not considered in this study.

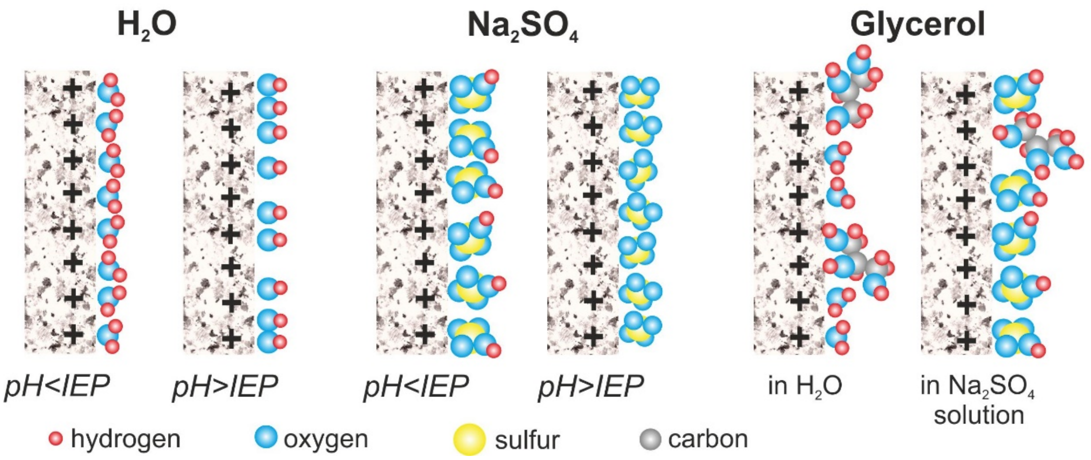

Figure S4 – Schematic representation of the BSO surface layer's composition in different liquids.

In glycerol solution, the zeta-potential became more positive. In this case, polyol can be adsorbed via the interaction with positive charged centres on the surface. Therefore, the functional groups of the polyol become potential-determining groups. Because glycerol does not dissociate (as a non-electrolyte) and its molecules are quite large, the negative surface charge of the BSO particles in glycerol solution decreased compared to BSO particles in water alone (Figure 5). This is because large glycerol molecules containing three OH groups cover the surface more sparsely than a denser layer of the functional groups obtained via interaction with small water molecules, or small ions from water, does (Figure 6).

The zeta-potential decreased when the samples were immersed in Na_2SO_4 solution, and the low IEP values might be connected with the surface's recharging, caused by the SO_4^{2-} anions being adsorbed on the positively charged surficial centres (Figure 6). The adsorbed ions became potential determining. In the aqueous solution, adsorbed sulfate-anions were present in a hydrolysed form according to the following reaction:

$$(=BiSO_4^-)_s + H_2O \leftrightarrow (=BiSO_4H)_s + OH^-$$

At a pH higher than the IEP, the surface is negatively charged due to the interaction with the OH^- ions:

$$(=BiSO_4H)_s + OH^- \leftrightarrow (=BiSO_4^-)_s + H_2O$$

At a pH lower than the IEP, the surface is positively charged due to the interaction with H^+ ions:

$$(=BiSO_4H)_s + H^+ \leftrightarrow (=BiSO_4H_2^+)_s$$

Or

$$=O_s + H^+ \leftrightarrow (=OH^+)_s$$

Therefore, the presence of large double-charged ions on the electrode's surface increased the C_{dl} for both Na_2SO_4 and Na_2SO_4 with glycerol joint solutions. Furthermore, the results obtained in the joint solution point at the competitive adsorption of Na_2SO_4 and glycerol when the sulfate-anions are in a leading position (Figure 6).

Section S5. Solution resistance study.

The equivalent circuit shown in Figure 4 (insets) simulated the processes in the studied systems. The R_s values for solution resistance were in the range of 20–130 Ω in the presence of the strong electrolyte. The calculated R_s (Table 6) was approximately equal to the Z' values in the high-frequency region of the Nyquist plot (Figure 4). The highest resistance was detected for BSO/TEOS material in the electrolyte alone solution. Here, we suggested that the difference in phase composition played its role: this sample was the only one that contained bismuth oxides (α- and β-, around 5%), whose conductivity is lower than that of bismuth silicates. So, Bi_2O_3 presence affected the measurements on the electrolyte/material interface providing the higher resistance value.

For Na_2SO_4 and glycerol joint solution we supposed that the glycerol molecules might block the semiconductor's surface, resulting in higher measured R_s values. However, R_s did not change for BSO/OH and BSO/NaSi in two liquids. And, unexpectedly, for BSO/TEOS the R_s for the joint Na_2SO_4 glycerol solution decreased compared to Na_2SO_4 alone. Adding glycerol to the water solution leads to a rearrangement of hydrogen bonds [11,12] that affects the proton-transfer phenomena. According to Behrends *et al.* [13], the glycerol-water mixtures exhibit a micro-heterogeneous nature. The existence of specific inter-molecular structures in such mixtures leads to relaxation phenomena in the bulk of the solution. As a result, the higher *AC* conductivity of glycerol-water mixtures can be observed [14]. Possibly, this is why for the BSO/TEOS sample, the presence of glycerol led to a significant decrease in the R_s value.

Section S6. Diffusion process at the SLI.

Moving on to the diffusion component of the electrochemical cell, it should be noted that the open circuit potential value in this study was determined by the oxygen and hydrogen evolution reactions (OER and HER, respectively). Thus, the Warburg component of the equivalent circuit (Figure 4) simulated the diffusion of the OER and HER products from the surface.

The parameter characterizing the diffusion's ideality (W_o-P) was basically lower than the required 0.5 value. This points to the deviation from the ideal diffusion due to the inhomogeneity of the electrode surface's composition and/or structure. Therefore, all of the studied electrodes demonstrated non-ideal diffusion behaviour.

The electrode surfaces consisted of BSO particles mixed with polystyrene. SEM images showed that the particle distribution in the polymer was not ideally homogenous. Hence, the electrodes' surfaces comprise two types of regions: dielectric polymer and semiconductor regions. This is the first degree of the inhomogeneity (structural inhomogeneity) that was characteristic of all of the samples. The second degree of inhomogeneity (structural as well) is from the non-homogeneous distribution of the BSO particles in their agglomerations, which differed from sample to sample. The third degree of inhomogeneity (compositional) is in how the phases in the particles are distributed (Bi_2SiO_5, $Bi_{12}SiO_{20}$, and two bismuth oxides for the BSO/TEOS sample). Thus, the deviation from the ideal diffusion is quite reasonable.

W_o-R and W_o-T are the two main parameters characterizing the species transport processes to/from the SLI. The diffusion resistance and time values in both solutions differed by one order of magnitude for BSO/TEOS and BSO/NaSi materials. And they differed for BSO/OH in the presence and absence of glycerol. This could be explained by the nature of glycerol molecules. As mentioned above, 0.05 M glycerol solution contains massive hydrated glycerol molecules, which cannot form a dense layer close to the semiconductor's surface. Therefore, the diffusion to the SLI was affected by the presence of large non-electrolyte molecules, even at a concentration lower than 10%. And from the Table 6 it is seen that the BSO/OH sample undergoes the glycerol adsorption influence stronger than other samples.

References

1. Al-Keisy, A. Ren, L.; Zheng, T.; Xu, X.; Higgins, M.; Hao, W.; Du, Y. Enhancement of charge separation in ferroelectric heterogeneous photocatalyst $Bi_4(SiO_4)_3$/Bi_2SiO_5 nanostructures, *Dalton Trans*. **2017**, *46*, 15582–15588. https://doi.org/10.1039/c7dt03193a.
2. Ding, S.; Xiong, X.; Liu, X.; Shi, Y.; Jiang, Q.; Hu, J. Synthesis and characterization of single-crystalline $Bi_2O_2SiO_3$ nanosheets with exposed {001} facets, *Catal. Sci. Technol*. **2017**, *7*, 3791–3801. https://doi.org/10.1039/c7cy01291h.

3. Ling, Y.; Dai, Y.; Direct Z-scheme hierarchical WO_3/BiOBr with enhanced photocatalytic degradation performance under visible light, *Appl. Surf. Sci.* **2020**, *509*, 145201. https://doi.org/10.1016/j.apsusc.2019.145201.

4. John, S.; Roy, S.C. CuO/Cu_2O nanoflake/nanowire heterostructure photocathode with enhanced surface area for photoelectrochemical solar energy conversion, *Appl. Surf. Sci.* **2020**, *509*, 144703. https://doi.org/10.1016/j.apsusc.2019.144703.

5. Bi, X.; Du, G.; Sun, D.; Zhang, M. Yu, Y.; Su, Q.; Ding, S.; Xu, B. Room-temperature synthesis of yellow TiO_2 nanoparticles with enhanced photocatalytic properties, *Appl. Surf. Sci.* **2020**, *511*, 145617. https://doi.org/10.1016/j.apsusc.2020.145617.

6. Dai, C.; Luo, H.; Li, J.; Du, C.; Liu, Z.; Yao, J. X-ray photoelectron spectroscopy and electrochemical investigation of the passive behavior of high-entropy $FeCoCrNiMo_x$ alloys in sulfuric acid, *Appl. Surf. Sci.* **2020**, *499*, 143903. https://doi.org/10.1016/j.apsusc.2019.143903.

7. Si, K.; Ma, J.; Lu, C.; Zhou, Y.; He, C.; Yang, D.; Wang, X.; Xu, X. A two-dimensional MoS_2/WSe_2 van der Waals heterostructure for enhanced photoelectric performance, *Appl. Surf. Sci.* **2020**, *507*, 145082. https://doi.org/10.1016/j.apsusc.2019.145082.

8. Bashiri, R.; Samsudin, M.F.R.; Mohamed, N.M.; Suhaimi, N.A.; Ling, L.Y.; Sufian, S.; Kait, C.F. Influence of growth time on photoelectrical characteristics and photocatalytic hydrogen production of decorated Fe_2O_3 on TiO_2 nanorod in photoelectrochemical cell, *Appl. Surf. Sci.* **2020**, *510*, 145482. https://doi.org/10.1016/j.apsusc.2020.145482.

9. Lopes, T.; Andrade, L.; Aguilar Ribeiro, H.; Mendes, A. Characterization of photoelectrochemical cells for water splitting by electrochemical impedance spectroscopy, *Int. J. Hydrogen Energy* **2010**, *35*, 11601–11608. https://doi.org/10.1016/j.ijhydene.2010.04.001.

10. Hwang, S. W.; Seo, D.H.; Kim, J. U.; Lee, D.K.; Choi, K.S.; Jeon, C.; Yu, H.K.; Cho, I.S. Bismuth vanadate photoanode synthesized by electron-beam evaporation of a single precursor source for enhanced solar water-splitting, *Appl. Surf. Sci.* **2020**, *528*, 146906. https://doi.org/10.1016/j.apsusc.2020.146906.

11. C. Chen, W. Li, Y. Song, J. Yang, Hydrogen bonding analysis of glycerol aqueous solutions: A molecular dynamics simulation study, J. Mol. Liq. 146 (2009) 23–28. https://doi.org/10.1016/j.molliq.2009.01.009.

12. T. Fisher, G. Zhou, Y. Shi, L. Huang, How does hydrogen bond network analysis reveal the golden ratio of water–glycerol mixtures? Phys.Chem.Chem.Phys. 22 (2020) https://doi.org/ 10.1039/c9cp06246g.

13. R. Behrends, K. Fuchs, U. Kaatze, Y. Hayashi, Y. Feldman, Dielectric properties of glycerol/water mixtures at temperatures between 10 and 50 ° C, J. Chem. Phys. 124 (2006). https://doi.org/10.1063/1.2188391.

14. P. Meaney, C. Fox, S. Geimer, K. Paulsen, Electrical Characterization of Glycerin: Water Mixtures: Implications for Use as a Coupling Medium in Microwave Tomography IEEE, Trans Microw Theory Tech. 65 (2017) 1471–1478. https://doi.org/10.1109/TMTT.2016.2638423.
